# Who Are Suitable Patients for Omitting Breast Surgery as an Exceptional Responder in Selected Molecular Subtypes of Breast Cancer After Neoadjuvant Systemic Treatment?

**DOI:** 10.3390/medicina61010048

**Published:** 2024-12-31

**Authors:** Ebru Sen, Mehmet Ali Nazlı, Göktürk Maralcan, Bekir Sıtkı Said Ulusoy, Mahmut Kaan Demircioğlu, Tuce Söylemez Akkurt, Mehmet Sökücü, Gökmen Umut Erdem, Mustafa Yıldırım

**Affiliations:** 1 Department of General Surgery, Başakşehir Çam and Sakura City Hospital İstanbul Türkiye, Istanbul 34480, Türkiye; 2 Interventional Radiology Section, Department of Radiology, Başakşehir Çam and Sakura City Hospital İstanbul Türkiye, Istanbul 34480, Türkiye; mnazli46@yahoo.com; 3 Department of General Surgery, Section of Endocrine and Breast Surgery, Sanko University Medical Faculty Gaziantep Türkiye, Gaziantep 27090, Türkiye; gokturkm2@gmail.com; 4 Section of Interventional Radiology, Department of Radiology, Sanko University Medical Faculty Gaziantep Türkiye, Gaziantep 27090, Türkiye; bsaidulusoy@hotmail.com; 5 Department of Surgical Oncology, Başakşehir Çam and Sakura City Hospital İstanbul Türkiye, İstanbul 34480, Türkiye; mahmutkaandemircioglu@gmail.com; 6 Department of Pathology, Başakşehir Çam and Sakura City Hospital İstanbul Türkiye, İstanbul 34480, Türkiye; tuce0791@gmail.com; 7 Department of Pathology, Sanko University Medical Faculty Gaziantep Türkiye, Gaziantep 27090, Türkiye; mehmet.sokucu@sankotip.com; 8 Department of Medical Oncology, Başakşehir Çam and Sakura City Hospital İstanbul Türkiye, İstanbul 34480, Türkiye; gokmenumut@hotmail.com; 9 Department of Medical Oncology, Sanko University Medical Faculty Gaziantep Türkiye, Gaziantep 27090, Türkiye; mustafayildirim7@yahoo.com

**Keywords:** neoadjuvant therapy, omission of surgery, postchemo-presurgical core biopsy

## Abstract

*Background and Objectives*: Among breast cancer molecular types, HER2 positive and triple negative (TN) subtypes have the highest likelihood of pathological complete response (pCR), which is a surrogate marker for reduced recurrence and improved patient survival after neoadjuvant systemic treatment (NST). Preoperative pathological identification of these exceptional responders is a new era. Therefore, we aimed to determine the accuracy of trucut biopsy in identifying the exceptional responders in selected molecular subtypes of breast cancer patients. *Materials and Methods*: This two-centre, observational, single-arm, prospective, pilot study was conducted between January and September 2024. The patients with TN or HER2 positive breast cancer whose breast tumour had completely disappeared on the radiological assessment including MRI after neoadjuvant therapy were enrolled. To assess neoadjuvant treatment response, a standardised biopsy protocol was used, consisting of 10 samples from the marked tumour area per patient by 12 G core needle. Then, all patients underwent surgery. The pathological results of both postchemo-presurgical biopsy and surgical breast specimen were compared. *Results*: The study included 20 patients. The mean age of the patients was 47.3 years. The median tumour size at diagnosis was 23.1 mm. All biopsy results were concordant with the findings of surgical specimen. Seventeen patients had a complete response. The remaining 3 patients had residual disease. *Conclusions*: Along with thorough patient selection, post-chemo radiological assessment and the reliable biopsy technique are the key points in accurately predicting response to neoadjuvant treatment. If an image-guided core biopsy confirms elimination of tumour tissue at the marked tumour area with a radiological complete response on MRI after NST in breast cancer patients with selected molecular subtypes, these may be suitable patients as exceptional responders in whom we can omit breast surgery.

## 1. Introduction

Neoadjuvant systemic therapy (NST) is a relevant part of the individualised breast cancer treatment, allowing for systemic and locoregional therapy to be tailored. Patients with a pathological complete response (pCR) after NST, namely exceptional responders, have been shown to have optimal outcomes with fewer recurrences and improved survival [1]. pCR is closely related to the molecular subtype of breast carcinoma. HER2-positive and triple-negative (TN) subtypes have the highest likelihood of pCR rates, exceeding 60% [2,3]. If it can be proven radiologically and pathologically that the breast tumour has been completely eliminated with NST, surgical excision may become unnecessary, especially for these types of tumours. Dynamic contrast-enhanced- (DCE) magnetic resonance imaging (MRI) before and after systemic treatment is accepted as the most accurate radiological method for monitoring response and estimating pCR, with a reported accuracy of 83% [4]. However, all imaging modalities, including PET-CT, are not satisfactory for predicting pCR following NST [5]. Therefore, current studies focus on the efficacy of image-guided minimally invasive biopsy (MIB) procedures of the clipped tumour bed after completion of systemic therapy to identify exceptional responders. MIB can be performed under ultrasonography, mammography, or MRI guidance. Based on various factors such as needle size and sampling mount, studies have shown the diverse false-negative rate (FNR) of MIB in detecting breast pCR to rank between 0 and 49% [6,7,8].

Thanks to NST, more conservative surgery has become possible in both the breast and axilla. Although segmental mastectomy has relatively low morbidity, surgical intervention still leads to trauma by impairing quality of life and cosmetic appearance [9]. Therefore, efforts to de-escalate the surgical treatment of breast cancer are ongoing.

In this pilot trial, we sought to evaluate the accuracy of postchemo-presurgical 12 G core needle biopsy in order to identify the exceptional responders in patients with breast cancer of selected molecular subtypes.

## 2. Materials and Methods

### 2.1. Study Design and Patient Selection

This multicentre, observational, single-arm, prospective pilot study was conducted at two centres in Turkey from January to September 2024. The study included women aged 26 years or older with invasive breast carcinoma of TN or hormone receptor-negative and HER2-positive type with unicentric stage I–III disease. Inclusion criteria were patients with complete clinical and complete radiological response at breast assessment after NST. Exclusion criteria were patients with a diagnosis of carcinoma in situ, metastatic disease, bilateral carcinoma, associated malignant microcalcifications, hormone receptor-positive tumour type, those who underwent upfront surgery, and those who had partial or no response to systemic treatment. The study was approved by the Çam Sakura City Hospital Institutional Review Board and Ethics Committee (Date: 27 December 2023-No:707). Prior to participation in the study, all patients were thoroughly informed that the postchemo-presurgical biopsy would not change the treatment strategy, and written informed consent was obtained.

Immunohistochemical oestrogen receptor (ER), progesterone receptor (PgR), and HER2 status had been evaluated according to the College of American Pathologists (CAP) Protocols. Positivity for HER2 had been defined as immunohistochemical positivity when >10% of tumour cells were stained intense and circumferentially. Equivocal HER2-positivity had been further evaluated by in situ hybridization according to ASCO/CAP guidelines. All patients had ultrasound, mammography (MG), and DCE-MRI examination before NST, and tumour beds were marked with a clip by the breast radiologist prior to systemic treatment. Suspicious axillary lymph nodes were examined by ultrasound-guided biopsy and, if metastatic, the most prominent node was also marked. In accordance with guidelines, patients with HER2-positive disease received four cycles of anthracycline and cyclophosphamide, subsequently followed by four cycles of taxane-based chemotherapy in combination with pertuzumab and trastuzumab. Alternatively, some patients received six cycles of carboplatin, docetaxel, pertuzumab, and trastuzumab. For patients with triple-negative disease, treatment involved an initial twelve-week course of carboplatin and paclitaxel chemotherapy, followed by four cycles of anthracycline and cyclophosphamide. Clinical follow-up was monthly.

From two to four weeks after the completion of neoadjuvant therapy, all patients underwent ultrasound and DCE-MRI to assess tumour response. Mammography was used in cases deemed necessary by the radiologist. Radiological complete response (rCR) in the breast was defined as focal distortion and disappearance of residual mass on ultrasound and complete absence of the mass and no contrast enhancement in the index tumour area on DCE-MRI.

### 2.2. Postchemo-Presurgical Core Biopsy and Surgery

In patients with clinical disappearance of the breast mass and radiological complete response, a biopsy of the index tumour was taken by the radiologist preoperatively a few days before surgery or intraoperatively just before the surgery, according to the preference of the patient and the surgeon. In a patient with high anxiety, the latter was chosen. Image-guided core needle biopsy (CNB) was performed under ultrasonography (USG) or mammography guidance depending on the visibility of the clipped tumour bed. The biopsy procedure was established according to a strict protocol: a 12-Gauge automated needle device and a 22 mm biopsy gun (Magnum Reusable Core Biopsy Instrument) were used to take samples near the marked tumour area without attempting to remove the clip. The standard number of cores removed was 10 for each patient. For preoperatively performed cases, mammography was performed after biopsy to ensure that there was no clip migration. Any procedure-related adverse events were recorded.

Surgical intervention was executed by the breast surgeon within 4–8 weeks after the last chemotherapy session, according to the MDT of each centre. Prior to surgery, the tumour bed was localised with a wire either ultrasonographically or stereotactically (Bard, DuaLok breast localisation wire, 15 cm). In addition, a topographical projection of the tumour bed was drawn on the skin. After resection, the specimen mammogram was performed to confirm the presence of the clip. Sentinel lymph node biopsy (SLNB) was used as a standard of care. If positive, axillary dissection was performed. In a patient with metastatic axillary node prior to NST that responded to therapy, targeted axillary dissection was applied.

The postchemo-presurgical biopsy and the surgical specimens were independently examined by a dedicated pathologist. Histopathological analysis of the biopsy was considered representative if it contained residual tumour cells or evidence of the previous tumour bed, characterised by the presence of stromal fibrosis, oedematous stroma with inflammatory cells, and macrophage infiltration. If the biopsy contained normal breast tissue or small inadequate material, it was accepted as an unrepresentative analysis. The absence of invasive and in situ carcinoma was considered a pathological complete response (pCR) of the breast, regardless of nodal status.

### 2.3. Statistical Analysis

Statistical analyses were performed using the SPSS version 27 (IBM Corp., Armonk, NY, USA) software package. Clinicopathological characteristics were reported descriptive statistics with absolute and relative frequencies (n, %) for categorical variables and median or range for continuous variables. Normality assumption of continuous variables was evaluated using the Shapiro–Wilk-W test. Power analysis was not performed, since this is a feasibility study that does not require sample size calculation [10]. Histopathological results of core biopsy were compared by those of surgical specimen. In order to determine the diagnostic performance of postchemo-presurgical core biopsy for the prediction of a pCR after NST, the accuracy, sensitivity, and specificity were calculated.

## 3. Results

### 3.1. Patient and Tumour Characteristics

A total of 20 patients with clinical and radiological complete response to treatment, aged between 26 and 69 years (median age 50 years), were included in this study. Molecularly, 12 patients had triple-negative disease, 8 patients had hormone-negative and HER2-positive tumours. Median initial tumour size was 20 mm, ranging from 8 to 45 mm. Nodal metastases were detected using FNA in eight patients. Five patients had N1 disease, and the rest had N2 disease. All tumours were invasive ductal carcinoma, except three; one of them was micropapillary carcinoma, another was mixed ductal and lobular carcinoma, and the third one was pleomorphic lobular carcinoma. Eighteen tumours were grade 3, and two were grade 2. The median Ki67 proliferation index was 50% (range 20–80%). Patient and tumour characteristics are presented in Table 1.

### 3.2. Postchemo-Presurgical Core Biopsy Procedure and Surgery

In 18 patients. the clipped tumour bed was visualised using ultrasound. The postchemo-presurgical biopsy was performed ultrasonographically in these patients. Biopsy was preoperatively applied under mammographic guidance in two patients. The procedure was performed preoperatively or intraoperatively in 13 and 7 patients, respectively. A biopsy-related adverse event was technical. In this patient, the tumour bed had to be re-clipped because the marker was accidentally removed during the biopsy. Another complication was patient discomfort, which was seen in two patients during the preoperative biopsy preparation. Therefore, biopsies were performed intraoperatively. Patient tolerance was otherwise excellent. There was no non-representative analysis. Details of the procedure are summarised in Table 2.

The tumour bed was localised preoperatively by wire under ultrasound in 18 patients and under mammography guidance in 2 patients, as well as by skin marking in all patients. All patients underwent breast-conserving surgery. The marker clip was documented intraoperatively by specimen graphs in all cases. After NST, SLNB or TAD were performed in 12 and 8 patients, respectively. Axillary dissection was added in two patients.

### 3.3. The Correlation of pCR Status

In three identical patients, residual disease was found in both the postchemo-presurgical biopsy and the surgical specimen. The remaining 17 patients had a complete response. The accuracy, sensitivity, and specificity of core biopsy for predicting a pCR after NST were 100% (Table 3).

Along with breast pathological response, concordant nodal pCR was seen in seven out of eight node-positive patients. In one patient, isolated tumour cells were detected in an axillary node.

Second-look evaluations were retrospectively performed on the radiological examinations of three patients who had no pCR. In one patient residual TN micropapillary carcinoma was detected on second-look MRI. The tumour located peripherally near the axilla could not be clearly visualised on post-NST MRI due to contrast enhancement and distortion of cardiac motion artefact. Another patient who had an HER2-positive tumour was initially evaluated as having a radiologically complete response. There was a thin band-like distortion in the tumour bed at ultrasound and vascularity could not be distinguished by a Doppler examination. But, low-contrast enhancement in the residual area was detected in DCE MRI in the second-look evaluation.

## 4. Discussion

Besides locally advanced breast cancer, neoadjuvant therapy is currently considered the first-line treatment for early-stage breast cancer, particularly in certain molecular subtypes [11,12]. Increased likelihood of breast-conserving surgery and decreased rates of axillary dissection, the unique in vivo response of patient’s tumour to chemotherapy, and ultimately pCR are considered target endpoints. After NST, surgery of the breast and axilla is an important part of the multidisciplinary treatment which determines the definitive pathological outcome in routine clinical practice. According to current guidelines, if the breast tumour has shrunk after NST, it is considered oncologically safe to remove residual breast tissue instead of the entire tumour area [13]. Therefore, if a complete pathological disappearance of breast carcinoma could be demonstrated following systemic therapy, the omission of surgery would be suggested as a reasonable approach in these exceptional responders. This approach has recently been challenged and can lead to a reduction in treatment costs, and, more importantly, a reduction in potential surgical complications [8,14,15]. A significant contribution can be provided by improving quality of life by eliminating the need for surgery in the pCR group.

Initial experiences prior to 2000, with the concept of avoiding surgery after systemic therapy in breast cancer patients, failed and were abandoned due to high rates of local recurrence [16,17]. Since both chemotherapeutic agents and the diagnostic tools to assess treatment response were far from their current counterparts, the impact of molecular subtypes on pCR was not known. Currently, the increasing use of improved neoadjuvant regimens together with targeted therapy and immunotherapy is associated with more patients with pCR [2,3]. Tumour subtypes are an indicator of the pattern of shrinkage as well as the rate of tumour response to NST [18]. The best responses with complete shrinkage to NST are seen in TN and HER2-positive subtypes, with reported rates of up to 70% [2,3]. However, luminal tumours respond poorly with scattering patterns like a honeycomb, which are thought to increase the sampling error of the biopsy should it respond well to NST [14].

Finding a reliable tool to predict pCR following NST has been a major topic in the literature [19]. Studies have highlighted the potential role of percutaneous image-guided MIB in predicting pCR [6]. Heil et al. from the University of Heidelberg first used vacuum-assisted biopsy (VAB) and core biopsy to investigate their ability to detect treatment response in breast cancer patients after NST and found a false-negative rate (FNR) of 49% [20]. The pCR rate of the cohort including all subtypes was 56%. They pointed out that MIB guided by a clip marker increased the rate of true-negative results. In the MİCRA trial of the Netherlands Cancer Institute, the FNR of ultrasound-guided core biopsy was found to be 37% in patients with radiological partial or complete response on MRI after NST [21]. Eight ultrasound-guided 14 G core biopsies were obtained preoperatively from the tumour marked at diagnosis. The pCR rate was 53%. These landmark trials have shed light on the importance of thorough patient selection and appropriate biopsy techniques in the context of MIB to identify the exceptional responders. Moreover, the selection of potential exceptional responders is related to the performance of post-chemotherapy imaging. Another pioneer study from MD Anderson Cancer Center reported a FNR of 5% and an accuracy of 98% of MIB in patients with TN-type and HER2-positive tumours with or without hormone-positive types and found a breast pCR of 47.5% in their feasibility trial [22]. Routine MRI examination was not performed in their study. In the present study, in order to reduce the non-complete response rate of the tumour, we restricted the inclusion of patients with TN or hormone-negative and HER2-positive subtypes in whom a complete response was demonstrated using MRI. In addition, patients with suspicious microcalcifications were considered an exclusion criterion in this cohort. Therefore, the pCR rate was 85%. Similarly, in a pilot study by the IEO from Milan the patients with TN and HER2-positive subtypes were involved. VAB had a pCR rate of 90% in patients with complete imaging response on either MRI or PET-CT [15]. They reported that a combination of MRI and PET-CT had superior performance than a combination of ultrasonography and mammography for the prediction of pCR. At our centre, we do not use PET-CT imaging after NST in the radiologically responsive group.

The accuracy of image-guided MIB is associated with the ability of adequate sampling of the residual tumour bed [8,23]. Among biopsy modalities, VAB has great performance by achieving faster acquisition of more tissue with a single insertion, giving almost equivalent results to open biopsy [15,23]. In the MD Anderson phase 2 trial related to elimination of breast surgery after NST, patient with <2 cm residual disease on post-NST imaging was accepted as an eligible candidate for biopsy. The biopsy procedure was defined as a minimum of 12 samples of 9 G VAB from the index area to detect exceptional responders [24]. However, not all institutions have the facilities to carry out VAB. In contrast, core biopsy is safe, simple, cost-effective, and highly accurate in expert hands [15]. In this cohort, to increase the quantity of tissue obtained, ten core samples were retrieved from each patient by using a 12 G needle, as a standardised protocol. With this technique, 100% accuracy was achieved. A similar result was reported by Lee et al. by obtaining at least five cores from the patient’s tumour with near pCR on MRI [25]. They also demonstrated no differences in the accuracy of US-guided biopsy between core biopsy via a 14 G needle and VAB via 10 G gun. These results support the planning of future trials as to surgical de-escalation after NST.

Second-look evaluations of radiological images from three patients with non-pCR demonstrated that residual tumours were missed in MRIs in two patients. Therefore, careful radiological assessment is a critical part of identifying exceptional responders. Fortunately, despite the misinterpretation of the radiological images, trucut biopsy of the index lesion accurately captured the residual tumour. One of the patients had micropapillary breast cancer, which is extremely rare and related to worse prognosis [26].

Although there is no universal guideline for the technique of postchemo-presurgical biopsy, ongoing trials are expected to clarify these details. In addition, this study found no difference in the effectiveness of MIB performed either preoperatively or intraoperatively. One of the disadvantages of the former was patient discomfort. In this trial, 2 out of 15 patients scheduled for preoperative biopsy underwent intraoperative biopsy for this reason. MIB is performed either by ultrasound, mammography, or MRI [23,27]. The latter is more complicated and requires the use of contrast media. As the biopsy procedure is operator-dependent, an experienced radiologist is required for satisfactory results regardless of the technique used [15]. As a new insight into efforts to accurately predict breast pCR, Pfob and colleagues have proposed the use of AI and developed multivariate algorithms that simultaneously incorporate tumour, patient, imaging, and VAB variables [28]. Considering the variety of methods and the validity of minimally invasive methods, we believe that our study contributes to the current literature with 100% accuracy of the trucut biopsy technique in identifying breast pCR. This technique, in the near future, may become a crucial part of the non-operative management of breast cancer patients after NST, particularly in the TN and pure HER2-positive groups.

Finally, the first results of omitting breast surgery in patients with no evidence of residual disease by VAB after NST have been reported from MD Anderson Cancer Center [24]. In 31 patients who omitted surgery followed by radiotherapy, no ipsilateral recurrence was detected at a median follow-up of 26 months. Similarly, the OPTIMIST trial from Korea has been launched, which is a prospective, multicentre, non-inferiority trial of 5-year DFS in patients with TN and HER2-positive breast cancer who have no residual tumour confirmed by image-guided biopsy following NST when breast surgery has been foregone [29]. Further consequences of these trials may lead to a radical revolution in breast cancer management by changing the therapeutic implications.

### Limitations

As a feasibility study, the analyses were limited by the small cohort size. It was difficult to persuade patients to undergo another biopsy before surgery, which did not affect their treatment. Since the procedure requires a dedicated radiologist, it should be planned in referral breast cancer centres in the context of a clinical trial. In addition, this strategy was associated with a limited number of breast cancer patients who had TN or HER2-positive subtypes, which accounted for 1/4 of breast cancers. It seems that the majority of breast cancer patients will not be suitable for this approach in the near future.

If larger series confirm our preliminary results, this MIB procedure may be a valid option to surgical resection in selected patients after NST.

## 5. Conclusions

In this study, we selected a group of patients who were not expected to have residual disease after NST because they were likely to have a high response to treatment. Thorough patient selection, careful evaluation of tumour response following systemic therapy, and a reliable biopsy technique are the key points for the achievement of pCR. Biopsy via 12 G needle with ten cores from the patients’ tumour with selected molecular subtypes of breast cancer who had a complete response to post-NST MRI showed whether there was a residual breast tumour, with an accuracy of 100%. Based on these results, a larger prospective multicentre clinical trial will be conducted. These studies are likely to pave the way for complete avoidance of breast surgery in exceptional responders.

## Figures and Tables

**Table 1:** Patient and tumour characteristics.

Characteristic		No (%)
Age	26–69 years (median 50 years)	
Molecular subtype	TN	12 (60%)
	HER2 +	8 (40%)
Median Tumour Size	8–45 mm (median 23.1 mm)	
Tumour stage	T1	11 (55%)
	T2	9 (45%)
Nodal stage	N0	12 (60%)
	N1	5 (25%)
	N2	3 (15%)
Histologic type	Invasive ductal carcinoma	17 (85%)
	Micropapillary carcinoma	1 (5%)
	Invasive pleomorphic lobular carcinoma	1 (5%)
	Mixed ductal and lobular carcinoma	1 (5%)
Histologic grade	G 1	0 (0%)
	G 2	2 (10%)
	G 3	18 (90%)
Ki67 proliferation index	20–80% (median 50%)	
Breast surgery	Breast-conserving surgery	20 (100%)
Axillary surgery	SLNB	12 (60%)
	TAD	6 (30%)
	AD	2 (10%)

**Table 2:** Details of the postchemo-presurgical core biopsy procedure.

Procedure		No (%)
Timing of CNB	Preoperatively	13 (65%)
	Intraoperatively	7 (35%)
Guidance for CNB	Ultrasound-guided biopsy	18 (90%)
	Mammography-guided biopsy	2 (10%)
Adverse event	Technical problem	1 (5%)
	Patient discomfort	2 (10%)
Biopsy representative	Yes	20 (100%)
	No	0 (0%)

**Table 3:** The correlation between pCR status between core biopsy and surgical specimen.

	Core Biopsy	Surgical Specimen
Complete response	17	17
Partial response	3	3
Total	20	20
Accuracy	100%	
Sensitivity	100%	
Specificity	100%	

## Data Availability

The original contributions presented in this study are included in the article material. Further inquiries can be directed to the corresponding author.
